# 1-{4-[Bis(4-fluoro­phen­yl)meth­yl]piperazin-1-yl}ethanone

**DOI:** 10.1107/S1600536812028097

**Published:** 2012-06-27

**Authors:** A. S. Dayananda, H. S. Yathirajan, Amanda C. Keeley, Jerry P. Jasinski

**Affiliations:** a Department of Studies in Chemistry, University of Mysore, Manasagangotri, Mysore 570 006, India; b Department of Chemistry, Keene State College, 229 Main Street, Keene, NH 03435-2001, USA

## Abstract

In the title compound, C_19_H_20_F_2_N_2_O, the six-membered piperazine group adopts a slightly distorted chair conformation. The dihedral angle between the mean planes of the two benzene rings is 73.4 (6)°. The mean plane of the ethanone group is twisted from the mean planes of the two benzene rings by 66.7 (8) and 86.2 (6)°. In the crystal, C—H⋯O and C—H⋯F inter­actions link the molecules, forming a three-dimensional structure.

## Related literature

For the biological activity of piperazines, see: Bogatcheva *et al.* (2006 ▶); Brockunier *et al.* (2004 ▶). For a review of pharmacological and toxicological information for piperazine derivatives, see: Elliott (2011 ▶). For related structures, see: Betz *et al.* (2011*a* ▶,*b* ▶); Dai *et al.* (2012 ▶); Dayananda *et al.* (2012*a* ▶,*b* ▶); Zhong *et al.* (2011 ▶). For puckering parameters, see: Cremer & Pople (1975 ▶). For reference bond-length data, see Allen *et al.* (1987 ▶).
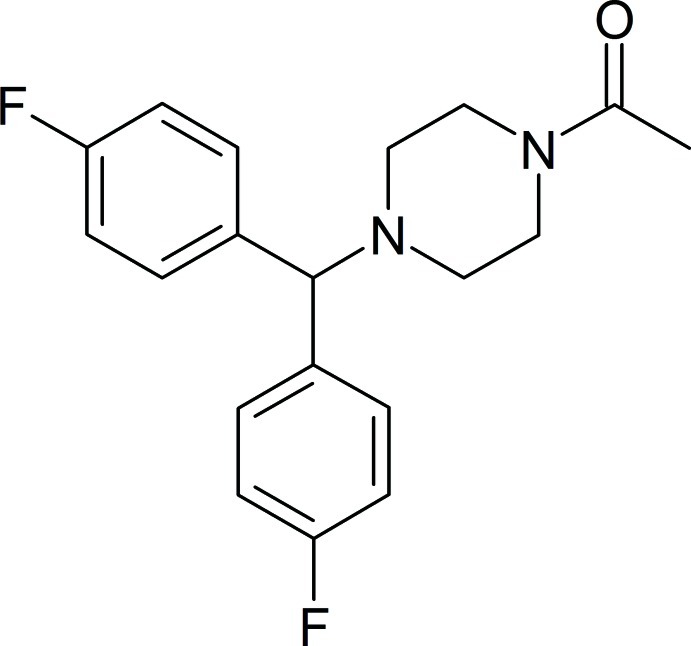


## Experimental

### 

#### Crystal data

C_19_H_20_F_2_N_2_O
*M* *_r_* = 330.37
Monoclinic, 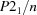

*a* = 10.1701 (5) Å
*b* = 16.5521 (5) Å
*c* = 11.1690 (5) Å
β = 114.690 (5)°
*V* = 1708.27 (14) Å^3^
*Z* = 4
Cu *K*α radiation
μ = 0.79 mm^−1^
*T* = 173 K
0.48 × 0.32 × 0.22 mm

#### Data collection

Oxford Xcalibur Eos Gemini diffractometer
Absorption correction: multi-scan (*CrysAlis RED*; Oxford Diffraction, 2010 ▶) *T*_min_ = 0.802, *T*_max_ = 1.000
10536 measured reflections
3297 independent reflections
2809 reflections with *I* > 2σ(*I*)
*R*_int_ = 0.026

#### Refinement

*R*[*F* ^2^ > 2σ(*F* ^2^)] = 0.054
*wR*(*F* ^2^) = 0.155
*S* = 1.04
3297 reflections
218 parameters
H-atom parameters constrained
Δρ_max_ = 0.41 e Å^−3^
Δρ_min_ = −0.34 e Å^−3^

### 

Data collection: *CrysAlis PRO* (Oxford Diffraction, 2010 ▶); cell refinement: *CrysAlis PRO*; data reduction: *CrysAlis RED* (Oxford Diffraction, 2010 ▶); program(s) used to solve structure: *SHELXS97* (Sheldrick, 2008 ▶); program(s) used to refine structure: *SHELXL97* (Sheldrick, 2008 ▶); molecular graphics: *SHELXTL* (Sheldrick, 2008 ▶); software used to prepare material for publication: *SHELXTL*.

## Supplementary Material

Crystal structure: contains datablock(s) global, I. DOI: 10.1107/S1600536812028097/zj2086sup1.cif

Structure factors: contains datablock(s) I. DOI: 10.1107/S1600536812028097/zj2086Isup2.hkl

Supplementary material file. DOI: 10.1107/S1600536812028097/zj2086Isup3.cml

Additional supplementary materials:  crystallographic information; 3D view; checkCIF report

## Figures and Tables

**Table 1:** Hydrogen-bond geometry (Å, °)

*D*—H⋯*A*	*D*—H	H⋯*A*	*D*⋯*A*	*D*—H⋯*A*
C13—H13⋯O1^i^	0.93	2.46	3.371 (2)	167
C15—H15⋯O1^i^	0.93	2.55	3.351 (3)	145
C18—H18⋯F2^ii^	0.93	2.54	3.319 (3)	142

Symmetry codes: (i) 
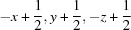
; (ii) 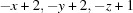.

**Fig. 1.**
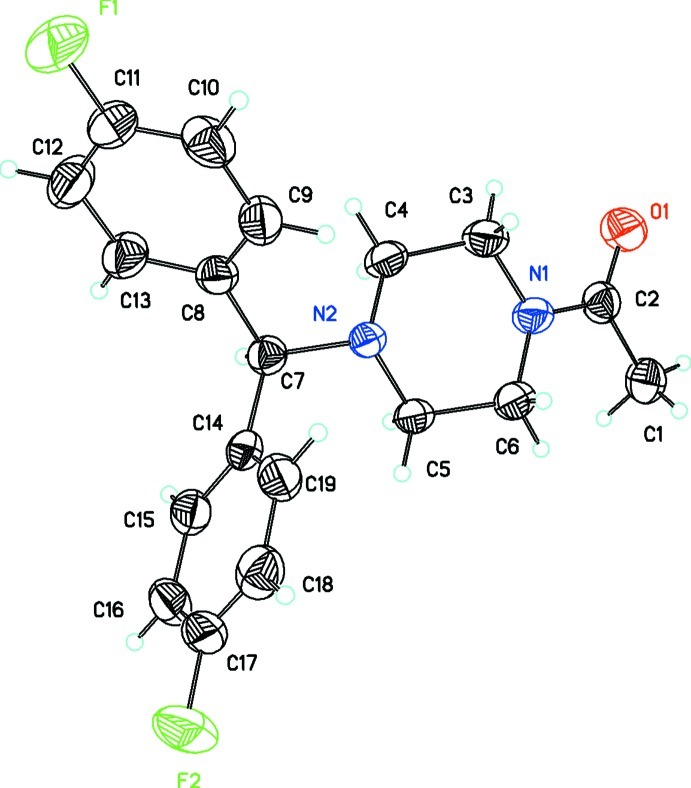
Molecular structure of the title compound showing the atom labeling scheme and 50% probability displacement ellipsoids.

**Fig. 2.**
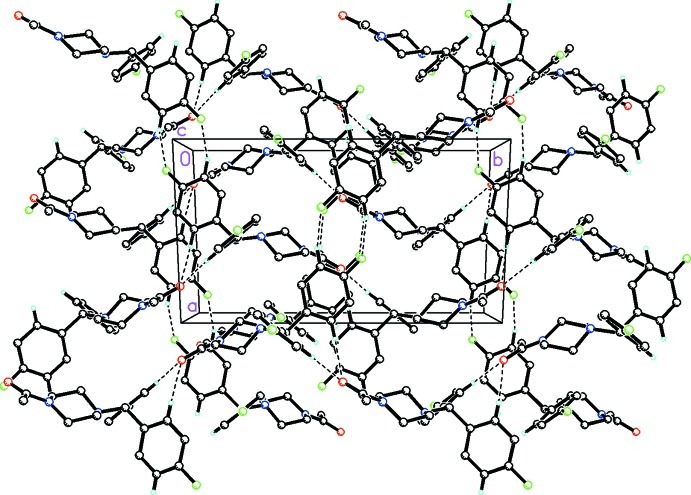
Packing diagram of the title compound viewed along the c axis. Dashed lines indicate weak C—H···O and C—H···F intermolecular interactions. The remaining H atoms have been removed for clarity.

## supplementary crystallographic information

### Comment

4,4'-Difluorobenzhydryl piperazine is an intermediate for the preparation of flunarizine which is a calcium channel blocker. Piperazines are among the most important building blocks in today's drug discovery and are found in biologically active compounds across a number of different therapeutic areas (Brockunier *et al.*, 2004; Bogatcheva *et al.*, 2006). A review on the current pharmacological and toxicological information for piperazine derivatives is described (Elliott, 2011).

The crystal structures of 4-[bis(4-fluorophenyl)methyl]piperazin-1-ium 2-(2-phenylethyl) benzoate (Betz *et al.*, 2011*a*), 4-[bis(4-fluorophenyl)methyl]piperazin-1-ium picrate (Betz *et al.*, 2011*b*), (*E*)-1-{4-[bis(4-fluorophenyl)methyl]piperazin-1-yl}-3-(4-ethoxyphenyl) prop-2-en-1-one (Zhong *et al.*, 2011), 4-[bis(4-fluorophenyl) methyl]piperazin-1-ium bis(trichloroacetate) 0.4-hydrate (Dayananda *et al.*, 2012*a*), 4-[bis(4-fluorophenyl)methyl] piperazin-1-ium 2-hydroxybenzoate 2-hydroxybenzoic acid monosolvate (Dayananda *et al.*, 2012*b*) and 1-[bis(4-fluorophenyl) methyl]-4-[2-(2-methylphenoxy)ethyl]piperazine (Dai *et al.*, 2012) have been reported. In the course of our studies on the salts of piperazines and in view of the importance of piperazines, this paper reports the crystal and molecular structure of the title compound, C_19_H_20_F_2_N_2_O, (I), which was accidentally obtained by the reaction of 4,4'-difluorobenzhydryl piperazine and acetyl salicylic acid.

In the asymmetric unit of the title compound, (I), the 6-membered piperazine group (N1/C3/C4/N2/C5/C6) adopts a slightly distorted chair conformation with puckering parameters Q, θ and φ of 0.568 (9) Å, 172.2 (7)°, and 350.979 (8)° (Cremer & Pople, 1975), respectively (Fig. 1). For an ideal chair θ has a value of 0 or 180°. Bond lengths are in normal ranges (Allen *et al.*, 1987). The dihedral angle between the mean planes of the two benzene rings is 73.4 (6)°. The mean plane of the ethanone group (C1/C2/O1/N1) is twisted from the mean planes of the two benzene rings by 66.7 (8)° and 86.2 (6)°. Weak C—H···O and C—H···F intermolecular interactions (Table 1) are observed providing increased stability with crystal packing (Fig. 2).

### Experimental

4,4'-Difluorobenzhydryl piperazine was obtained from R. L. Fine Chem., Bengaluru, India. 4,4'-Difluorobenzhydryl piperazine (2.88 g, 0.01 mol) was dissolved in 10 ml of absolute ethanol and acetylsalicylic acid (1.81 g, 0.01 mol) was also dissolved in 10 ml of absolute ethanol. Both the solutions were mixed and stirred in a beaker at 333 K for 30 min. The mixture was kept aside for a day at room temperature. The compound formed was filtered and dried in a vacuum desiccator over phosphorous pentoxide. The compound was recrystallized from a mixture of toluene and dimethyl formamide by slow evaporation (m.p. 418–423 K).

### Refinement

All of the H atoms were placed in their calculated positions and then refined using the riding model with C—H lengths of 0.93, 0.98 (CH) or 0.96 Å (CH_3_). The isotropic displacement parameters for these atoms were set to 1.19 to 1.20 (CH), 1.19 to 1.20 (CH_2_) or 1.50 (CH_3_) times *U*_eq_ of the parent atom.

### Crystal data

**Table:**

C_19_H_20_F_2_N_2_O	*F*(000) = 696
*M**_r_* = 330.37	*D*_x_ = 1.285 Mg m^−^^3^
Monoclinic, *P*2_1_/*n*	Cu *K*α radiation, λ = 1.54178 Å
Hall symbol: -P 2yn	Cell parameters from 4103 reflections
*a* = 10.1701 (5) Å	θ = 4.4–71.2°
*b* = 16.5521 (5) Å	µ = 0.79 mm^−^^1^
*c* = 11.1690 (5) Å	*T* = 173 K
β = 114.690 (5)°	Chunk, colourless
*V* = 1708.27 (14) Å^3^	0.48 × 0.32 × 0.22 mm
*Z* = 4	

### Data collection

**Table:**

Oxford Xcalibur Eos Gemini diffractometer	3297 independent reflections
Radiation source: Enhance (Cu) X-ray Source	2809 reflections with *I* > 2σ(*I*)
Graphite monochromator	*R*_int_ = 0.026
Detector resolution: 16.1500 pixels mm^-1^	θ_max_ = 71.4°, θ_min_ = 5.0°
ω scans	*h* = −12→12
Absorption correction: multi-scan (*CrysAlis RED*; Oxford Diffraction, 2010)	*k* = −12→20
*T*_min_ = 0.802, *T*_max_ = 1.000	*l* = −13→13
10536 measured reflections	

### Refinement

**Table:**

Refinement on *F*^2^	Primary atom site location: structure-invariant direct methods
Least-squares matrix: full	Secondary atom site location: difference Fourier map
*R*[*F*^2^ > 2σ(*F*^2^)] = 0.054	Hydrogen site location: inferred from neighbouring sites
*wR*(*F*^2^) = 0.155	H-atom parameters constrained
*S* = 1.04	*w* = 1/[σ^2^(*F*_o_^2^) + (0.0781*P*)^2^ + 0.7598*P*] where *P* = (*F*_o_^2^ + 2*F*_c_^2^)/3
3297 reflections	(Δ/σ)_max_ < 0.001
218 parameters	Δρ_max_ = 0.41 e Å^−^^3^
0 restraints	Δρ_min_ = −0.34 e Å^−^^3^

### Special details

**Table:**

Geometry. All e.s.d.'s (except the e.s.d. in the dihedral angle between two l.s. planes) are estimated using the full covariance matrix. The cell e.s.d.'s are taken into account individually in the estimation of e.s.d.'s in distances, angles and torsion angles; correlations between e.s.d.'s in cell parameters are only used when they are defined by crystal symmetry. An approximate (isotropic) treatment of cell e.s.d.'s is used for estimating e.s.d.'s involving l.s. planes.
Refinement. Refinement of *F*^2^ against ALL reflections. The weighted *R*-factor *wR* and goodness of fit *S* are based on *F*^2^, conventional *R*-factors *R* are based on *F*, with *F* set to zero for negative *F*^2^. The threshold expression of *F*^2^ > σ(*F*^2^) is used only for calculating *R*-factors(gt) *etc*. and is not relevant to the choice of reflections for refinement. *R*-factors based on *F*^2^ are statistically about twice as large as those based on *F*, and *R*-factors based on ALL data will be even larger.

### Fractional atomic coordinates and isotropic or equivalent isotropic displacement parameters (Å^2^)

**Table:**

	*x*	*y*	*z*	*U*_iso_*/*U*_eq_	
F1	0.5160 (2)	0.80482 (10)	−0.24955 (14)	0.0811 (5)	
F2	0.86017 (18)	1.06144 (9)	0.55722 (15)	0.0788 (5)	
O1	0.29530 (17)	0.50309 (9)	0.41107 (15)	0.0534 (4)	
N1	0.40533 (18)	0.62266 (10)	0.42534 (15)	0.0410 (4)	
N2	0.48228 (15)	0.75448 (8)	0.30161 (13)	0.0304 (3)	
C1	0.3741 (3)	0.57161 (13)	0.6169 (2)	0.0520 (5)	
H1A	0.3212	0.5298	0.6371	0.078*	
H1B	0.3382	0.6234	0.6280	0.078*	
H1C	0.4749	0.5674	0.6752	0.078*	
C2	0.3555 (2)	0.56254 (11)	0.47665 (19)	0.0395 (4)	
C3	0.3933 (2)	0.61611 (12)	0.29096 (19)	0.0465 (5)	
H3A	0.3142	0.5801	0.2406	0.056*	
H3B	0.4819	0.5935	0.2922	0.056*	
C4	0.3666 (2)	0.69801 (12)	0.22666 (17)	0.0406 (4)	
H4A	0.3605	0.6930	0.1380	0.049*	
H4B	0.2749	0.7189	0.2206	0.049*	
C5	0.4817 (2)	0.76336 (11)	0.43201 (17)	0.0363 (4)	
H5A	0.3886	0.7842	0.4222	0.044*	
H5B	0.5555	0.8019	0.4835	0.044*	
C6	0.5101 (2)	0.68390 (12)	0.50348 (18)	0.0426 (5)	
H6A	0.6072	0.6658	0.5213	0.051*	
H6B	0.5038	0.6909	0.5872	0.051*	
C7	0.46380 (18)	0.83174 (11)	0.23205 (16)	0.0333 (4)	
H7	0.3663	0.8519	0.2120	0.040*	
C8	0.47860 (19)	0.82157 (11)	0.10209 (17)	0.0356 (4)	
C9	0.5661 (2)	0.76331 (12)	0.0844 (2)	0.0430 (4)	
H9	0.6165	0.7276	0.1526	0.052*	
C10	0.5799 (2)	0.75726 (12)	−0.0344 (2)	0.0508 (5)	
H10	0.6388	0.7181	−0.0465	0.061*	
C11	0.5044 (3)	0.81058 (13)	−0.1324 (2)	0.0522 (5)	
C12	0.4182 (3)	0.86953 (15)	−0.1176 (2)	0.0576 (6)	
H12	0.3689	0.9055	−0.1856	0.069*	
C13	0.4060 (2)	0.87437 (13)	0.00127 (19)	0.0472 (5)	
H13	0.3477	0.9141	0.0129	0.057*	
C14	0.5726 (2)	0.89431 (11)	0.31739 (16)	0.0349 (4)	
C15	0.5244 (2)	0.96762 (12)	0.34201 (19)	0.0426 (4)	
H15	0.4259	0.9791	0.3044	0.051*	
C16	0.6209 (3)	1.02416 (12)	0.4218 (2)	0.0522 (5)	
H16	0.5883	1.0736	0.4384	0.063*	
C17	0.7645 (3)	1.00628 (13)	0.4757 (2)	0.0507 (5)	
C18	0.8181 (2)	0.93527 (13)	0.4525 (2)	0.0496 (5)	
H18	0.9171	0.9251	0.4897	0.060*	
C19	0.7207 (2)	0.87896 (12)	0.37212 (19)	0.0419 (4)	
H19	0.7546	0.8303	0.3544	0.050*	

### Atomic displacement parameters (Å^2^)

**Table:**

	*U*^11^	*U*^22^	*U*^33^	*U*^12^	*U*^13^	*U*^23^
F1	0.1304 (14)	0.0839 (11)	0.0563 (9)	0.0159 (10)	0.0658 (9)	0.0004 (7)
F2	0.0951 (11)	0.0602 (9)	0.0724 (10)	−0.0414 (8)	0.0264 (8)	−0.0204 (7)
O1	0.0659 (9)	0.0428 (8)	0.0519 (8)	−0.0214 (7)	0.0250 (7)	−0.0029 (6)
N1	0.0530 (9)	0.0380 (8)	0.0314 (8)	−0.0133 (7)	0.0170 (7)	−0.0030 (6)
N2	0.0345 (7)	0.0299 (7)	0.0268 (7)	−0.0034 (6)	0.0128 (6)	−0.0012 (5)
C1	0.0722 (14)	0.0438 (11)	0.0454 (11)	−0.0056 (10)	0.0300 (10)	0.0073 (9)
C2	0.0417 (9)	0.0368 (10)	0.0405 (10)	−0.0024 (8)	0.0177 (8)	0.0035 (8)
C3	0.0651 (12)	0.0400 (10)	0.0374 (10)	−0.0168 (9)	0.0245 (9)	−0.0084 (8)
C4	0.0477 (10)	0.0448 (10)	0.0278 (8)	−0.0129 (8)	0.0143 (8)	−0.0043 (7)
C5	0.0448 (10)	0.0348 (9)	0.0278 (9)	−0.0085 (7)	0.0137 (7)	−0.0037 (7)
C6	0.0516 (11)	0.0410 (10)	0.0305 (9)	−0.0107 (8)	0.0126 (8)	−0.0011 (7)
C7	0.0355 (8)	0.0342 (9)	0.0315 (9)	0.0023 (7)	0.0152 (7)	0.0004 (7)
C8	0.0410 (9)	0.0347 (9)	0.0334 (9)	−0.0048 (7)	0.0181 (7)	−0.0038 (7)
C9	0.0492 (11)	0.0379 (10)	0.0447 (11)	0.0022 (8)	0.0224 (9)	0.0050 (8)
C10	0.0624 (13)	0.0402 (11)	0.0652 (14)	0.0035 (9)	0.0420 (11)	−0.0045 (9)
C11	0.0776 (14)	0.0523 (12)	0.0429 (11)	0.0011 (11)	0.0411 (11)	−0.0009 (9)
C12	0.0765 (15)	0.0617 (14)	0.0387 (11)	0.0184 (12)	0.0281 (11)	0.0106 (10)
C13	0.0594 (12)	0.0488 (11)	0.0398 (10)	0.0111 (9)	0.0271 (9)	0.0023 (8)
C14	0.0449 (9)	0.0345 (9)	0.0265 (8)	−0.0055 (7)	0.0161 (7)	0.0033 (7)
C15	0.0501 (11)	0.0404 (10)	0.0392 (10)	0.0029 (8)	0.0206 (8)	0.0048 (8)
C16	0.0821 (16)	0.0304 (10)	0.0503 (12)	−0.0004 (10)	0.0338 (11)	−0.0008 (8)
C17	0.0689 (14)	0.0413 (11)	0.0399 (10)	−0.0249 (10)	0.0207 (10)	−0.0024 (8)
C18	0.0448 (10)	0.0541 (12)	0.0472 (11)	−0.0080 (9)	0.0165 (9)	0.0082 (9)
C19	0.0500 (11)	0.0349 (10)	0.0442 (10)	0.0001 (8)	0.0232 (9)	0.0015 (8)

### Geometric parameters (Å, º)

**Table:**

F1—C11	1.364 (2)	C7—C14	1.524 (2)
F2—C17	1.367 (2)	C7—C8	1.530 (2)
O1—C2	1.227 (2)	C7—H7	0.9800
N1—C2	1.349 (2)	C8—C13	1.372 (3)
N1—C3	1.458 (2)	C8—C9	1.381 (3)
N1—C6	1.465 (2)	C9—C10	1.396 (3)
N2—C4	1.462 (2)	C9—H9	0.9300
N2—C5	1.466 (2)	C10—C11	1.365 (3)
N2—C7	1.467 (2)	C10—H10	0.9300
C1—C2	1.505 (3)	C11—C12	1.368 (3)
C1—H1A	0.9600	C12—C13	1.386 (3)
C1—H1B	0.9600	C12—H12	0.9300
C1—H1C	0.9600	C13—H13	0.9300
C3—C4	1.505 (3)	C14—C15	1.378 (3)
C3—H3A	0.9700	C14—C19	1.393 (3)
C3—H3B	0.9700	C15—C16	1.379 (3)
C4—H4A	0.9700	C15—H15	0.9300
C4—H4B	0.9700	C16—C17	1.359 (3)
C5—C6	1.503 (3)	C16—H16	0.9300
C5—H5A	0.9700	C17—C18	1.365 (3)
C5—H5B	0.9700	C18—C19	1.383 (3)
C6—H6A	0.9700	C18—H18	0.9300
C6—H6B	0.9700	C19—H19	0.9300
			
C2—N1—C3	119.85 (15)	C14—C7—C8	109.55 (14)
C2—N1—C6	124.48 (15)	N2—C7—H7	108.2
C3—N1—C6	113.10 (15)	C14—C7—H7	108.2
C4—N2—C5	107.26 (13)	C8—C7—H7	108.2
C4—N2—C7	111.08 (13)	C13—C8—C9	119.04 (17)
C5—N2—C7	112.70 (13)	C13—C8—C7	118.44 (16)
C2—C1—H1A	109.5	C9—C8—C7	122.48 (16)
C2—C1—H1B	109.5	C8—C9—C10	120.84 (18)
H1A—C1—H1B	109.5	C8—C9—H9	119.6
C2—C1—H1C	109.5	C10—C9—H9	119.6
H1A—C1—H1C	109.5	C11—C10—C9	118.04 (18)
H1B—C1—H1C	109.5	C11—C10—H10	121.0
O1—C2—N1	121.36 (17)	C9—C10—H10	121.0
O1—C2—C1	121.08 (17)	F1—C11—C10	118.60 (19)
N1—C2—C1	117.55 (16)	F1—C11—C12	118.8 (2)
N1—C3—C4	110.18 (16)	C10—C11—C12	122.56 (19)
N1—C3—H3A	109.6	C11—C12—C13	118.4 (2)
C4—C3—H3A	109.6	C11—C12—H12	120.8
N1—C3—H3B	109.6	C13—C12—H12	120.8
C4—C3—H3B	109.6	C8—C13—C12	121.12 (19)
H3A—C3—H3B	108.1	C8—C13—H13	119.4
N2—C4—C3	111.16 (15)	C12—C13—H13	119.4
N2—C4—H4A	109.4	C15—C14—C19	118.84 (17)
C3—C4—H4A	109.4	C15—C14—C7	119.82 (17)
N2—C4—H4B	109.4	C19—C14—C7	121.33 (16)
C3—C4—H4B	109.4	C14—C15—C16	120.7 (2)
H4A—C4—H4B	108.0	C14—C15—H15	119.7
N2—C5—C6	111.14 (15)	C16—C15—H15	119.7
N2—C5—H5A	109.4	C17—C16—C15	118.8 (2)
C6—C5—H5A	109.4	C17—C16—H16	120.6
N2—C5—H5B	109.4	C15—C16—H16	120.6
C6—C5—H5B	109.4	C16—C17—C18	122.94 (19)
H5A—C5—H5B	108.0	C16—C17—F2	118.9 (2)
N1—C6—C5	111.02 (15)	C18—C17—F2	118.1 (2)
N1—C6—H6A	109.4	C17—C18—C19	118.0 (2)
C5—C6—H6A	109.4	C17—C18—H18	121.0
N1—C6—H6B	109.4	C19—C18—H18	121.0
C5—C6—H6B	109.4	C18—C19—C14	120.79 (19)
H6A—C6—H6B	108.0	C18—C19—H19	119.6
N2—C7—C14	111.23 (13)	C14—C19—H19	119.6
N2—C7—C8	111.40 (14)		
			
C3—N1—C2—O1	−2.5 (3)	C7—C8—C9—C10	−178.25 (18)
C6—N1—C2—O1	−163.00 (19)	C8—C9—C10—C11	0.0 (3)
C3—N1—C2—C1	178.49 (18)	C9—C10—C11—F1	−179.4 (2)
C6—N1—C2—C1	17.9 (3)	C9—C10—C11—C12	0.8 (4)
C2—N1—C3—C4	145.48 (18)	F1—C11—C12—C13	179.4 (2)
C6—N1—C3—C4	−51.9 (2)	C10—C11—C12—C13	−0.8 (4)
C5—N2—C4—C3	−62.16 (19)	C9—C8—C13—C12	0.6 (3)
C7—N2—C4—C3	174.28 (15)	C7—C8—C13—C12	178.3 (2)
N1—C3—C4—N2	58.1 (2)	C11—C12—C13—C8	0.1 (4)
C4—N2—C5—C6	60.94 (19)	N2—C7—C14—C15	−124.60 (17)
C7—N2—C5—C6	−176.50 (14)	C8—C7—C14—C15	111.80 (18)
C2—N1—C6—C5	−147.07 (18)	N2—C7—C14—C19	55.1 (2)
C3—N1—C6—C5	51.2 (2)	C8—C7—C14—C19	−68.5 (2)
N2—C5—C6—N1	−56.0 (2)	C19—C14—C15—C16	−1.4 (3)
C4—N2—C7—C14	172.68 (14)	C7—C14—C15—C16	178.28 (17)
C5—N2—C7—C14	52.28 (18)	C14—C15—C16—C17	0.0 (3)
C4—N2—C7—C8	−64.78 (18)	C15—C16—C17—C18	1.3 (3)
C5—N2—C7—C8	174.82 (14)	C15—C16—C17—F2	−178.55 (18)
N2—C7—C8—C13	151.90 (17)	C16—C17—C18—C19	−1.1 (3)
C14—C7—C8—C13	−84.6 (2)	F2—C17—C18—C19	178.75 (18)
N2—C7—C8—C9	−30.5 (2)	C17—C18—C19—C14	−0.4 (3)
C14—C7—C8—C9	93.0 (2)	C15—C14—C19—C18	1.6 (3)
C13—C8—C9—C10	−0.7 (3)	C7—C14—C19—C18	−178.09 (16)

### Hydrogen-bond geometry (Å, º)

**Table:**

*D*—H···*A*	*D*—H	H···*A*	*D*···*A*	*D*—H···*A*
C13—H13···O1^i^	0.93	2.46	3.371 (2)	167
C15—H15···O1^i^	0.93	2.55	3.351 (3)	145
C18—H18···F2^ii^	0.93	2.54	3.319 (3)	142

Symmetry codes: (i) −*x*+1/2, *y*+1/2, −*z*+1/2; (ii) −*x*+2, −*y*+2, −*z*+1.

## References

[bb1] Allen, F. H., Kennard, O., Watson, D. G., Brammer, L., Orpen, A. G. & Taylor, R. (1987). * J. Chem. Soc. Perkin Trans. 2*, pp. S1–19.

[bb2] Betz, R., Gerber, T., Hosten, E., Dayananda, A. S. & Yathirajan, H. S. (2011*a*). *Acta Cryst.* E**67**, o2783–o2784. 10.1107/S160053681103902X PMC3201291 22064768

[bb3] Betz, R., Gerber, T., Hosten, E., Dayananda, A. S., Yathirajan, H. S. & Narayana, B. (2011*b*). *Acta Cryst.* E**67**, o2587–o2588. 10.1107/S160053681103580X PMC3201484 22064439

[bb4] Bogatcheva, E., Hanrahan, C., Nikonenko, B., Samala, R., Chen, P., Gearhart, J., Barbosa, F., Einck, L., Nacy, C. A. & Protopopova, M. (2006). *J. Med. Chem.* **49**, 3045–3048. 10.1021/jm050948+ PMC4869334 16722620

[bb5] Brockunier, L. L., He, J., Colwell, L. F. Jr, Habulihaz, B., He, H., Leiting, B., Lyons, K. A., Marsilio, F., Patel, R. A., Teffera, Y., Wu, J. K., Thornberry, N. A., Weber, A. E. & Parmee, E. R. (2004). *Bioorg. Med. Chem. Lett.* **14**, 4763–4766. 10.1016/j.bmcl.2004.06.065 15324904

[bb6] Cremer, D. & Pople, J. A. (1975). *J. Am. Chem. Soc.* **97**, 1354–1358.

[bb7] Dai, Z.-H., Zhong, Y. & Wu, B. (2012). *Acta Cryst.* E**68**, o1077. 10.1107/S1600536812010744 PMC3344032 22589941

[bb8] Dayananda, A. S., Yathirajan, H. S. & Flörke, U. (2012*a*). *Acta Cryst.* E**68**, o968. 10.1107/S1600536812009282 PMC3343943 22590024

[bb9] Dayananda, A. S., Yathirajan, H. S. & Flörke, U. (2012*b*). *Acta Cryst.* E**68**, o1180. 10.1107/S1600536812012329 PMC3344118 22606121

[bb10] Elliott, S. (2011). Drug Test Anal. 3, 430–438. 10.1002/dta.307 21744514

[bb11] Oxford Diffraction (2010). *CrysAlis PRO* and *CrysAlis RED* Oxford Diffraction Ltd, Yarnton, Oxfordshire, England.

[bb12] Sheldrick, G. M. (2008). *Acta Cryst.* A**64**, 112–122. 10.1107/S0108767307043930 18156677

[bb13] Zhong, Y., Zhang, X. P. & Wu, B. (2011). *Acta Cryst.* E**67**, o3342. 10.1107/S160053681104801X PMC3238988 22199837

